# Characterization of the *Six1 *homeobox gene in normal mammary gland morphogenesis

**DOI:** 10.1186/1471-213X-10-4

**Published:** 2010-01-14

**Authors:** Ricardo D Coletta, Erica L McCoy, Valerie Burns, Kiyoshi Kawakami, James L McManaman, John J Wysolmerski, Heide L Ford

**Affiliations:** 1 Department of Obstetrics and Gynecology, University of Colorado Denver, Anschutz Medical Campus, 12800 E. 19th Ave., Aurora, CO, 80045, USA; 2 Program in Molecular Biology, University of Colorado Denver, Anschutz Medical Campus, 12800 E. 19th Ave., Aurora, CO, 80045, USA; 3 Departments of Physiology and Biophysics, University of Colorado Denver, Anschutz Medical Campus, 12800 E. 19th Ave., Aurora, CO, 80045, USA; 4 Biochemistry and Molecular Genetics, University of Colorado Denver, Anschutz Medical Campus, 12800 E. 19th Ave., Aurora, CO, 80045, USA; 5 Department of Oral Diagnosis, State University of Campinas, School of Dentistry, Av. Limeira, 901 Caixa Postal 52, Piracicaba, SP, CEP 13414-903, Brazil; 6 Division of Biology, Center for Molecular Medicine, Jichi Medical University, 3311-1 Yakushiji, Shimotsuke, Tochigi, 329-0498, Japan; 7 Division of Endocrinology and Metabolism, Department of Internal Medicine, Yale University School of Medicine, PO Box 208020, New Haven, CT 06520-8020, USA

## Abstract

**Background:**

The *Six1 *homeobox gene is highly expressed in the embryonic mammary gland, continues to be expressed in early postnatal mammary development, but is lost when the mammary gland differentiates during pregnancy. However, *Six1 *is re-expressed in breast cancers, suggesting that its re-instatement in the adult mammary gland may contribute to breast tumorigenesis via initiating a developmental process out of context. Indeed, recent studies demonstrate that Six1 overexpression in the adult mouse mammary gland is sufficient for initiating invasive carcinomas, and that its overexpression in xenograft models of mammary cancer leads to metastasis. These data demonstrate that Six1 is causally involved in both breast tumorigenesis and metastasis, thus raising the possibility that it may be a viable therapeutic target. However, because Six1 is highly expressed in the developing mammary gland, and because it has been implicated in the expansion of mammary stem cells, targeting Six1 as an anti-cancer therapy may have unwanted side effects in the breast.

**Results:**

We sought to determine the role of Six1 in mammary development using two independent mouse models. To study the effect of Six1 loss in early mammary development when Six1 is normally expressed, *Six1*^-/- ^embryonic mammary glands were transplanted into *Rag1*^-/- ^mice. In addition, to determine whether Six1 downregulation is required during later stages of development to allow for proper differentiation, we overexpressed Six1 during adulthood using an inducible, mammary-specific transgenic mouse model. Morphogenesis of the mammary gland occurred normally in animals transplanted with *Six1*^-/- ^embryonic mammary glands, likely through the redundant functions of other Six family members such as *Six2 *and *Six4*, whose expression was increased in response to Six1 loss. Surprisingly, inappropriate expression of Six1 in the adult mammary gland, when levels are normally low to absent, did not inhibit normal mammary differentiation during pregnancy or lactation.

**Conclusions:**

Six1 is not critical for normal mammary gland development, since neither loss nor inappropriate overexpression of Six1 adversely affects normal mammary gland development or function. However, as both *Six2 *and *Six4 *levels are increased in *Six1*^-/- ^mammary glands, we postulate that these Six family members are functionally redundant in the gland, as is true of many homeobox gene families. This data, in conjunction with recent findings that Six1 is capable of promoting breast cancer initiation and progression, suggest that Six1 may serve as a reasonable chemotherapeutic target in a clinical setting, particularly for those women diagnosed with breast cancer in their childbearing years.

## Background

Homeodomain-containing transcription factors are "master regulators" of normal development, controlling important cellular mechanisms, such as proliferation, differentiation, apoptosis, cell shape, cell adhesion and migration [1]. In addition to their important roles in normal development, numerous homeobox genes are misexpressed in both solid tumors [2,3] and hematopoietic malignancies [4], where it is thought that they impact neoplastic disease by reinitiating their developmental programs [1].

The *Six *family (*Six1-6*) of homeobox genes are important developmental regulators that are frequently misexpressed in cancers [5]. In the majority of cases studied, members of the Six family promote expansion of progenitor cell populations prior to differentiation, primarily through pro-proliferative and pro-survival mechanisms [6-9]. The most extensively studied Six family member in cancer is Six1, which regulates proliferation, survival, migration and invasion in both a developmental and tumorigenic context [10-13]. Six1 is broadly expressed in the developing embryo, in organs such as the kidney [14-16], muscle [15-17], sensory structures [14,18-20], and craniofacial structures arising from Rathke's pouch, including the pituitary, thyroid, and parathyroid glands, head muscles, facial skeleton, salivary glands, and cranial nerves [14,21]. Given the broad expression pattern of Six1 during embryogenesis, it is no surprise that its loss results in a variety of profound defects. For example, the *Six1 *knockout mouse dies shortly after birth due to hypoplasia of the diaphragm skeletal muscle, which inhibits its ability to breathe [17]. In addition to muscle hypoplasia and thoracic skeletal defects that are found throughout the *Six1 *knockout mouse [17], defects in other organs are observed, including the kidney, thymus, and craniofacial structures such as the inner, outer and middle ear, the olfactory epithelium, the craniofacial skeleton, and the lacrimal and salivary glands [8,14-17,19,22]. Interestingly, the majority of *Six1 *knockout defects are due to a reduction in proliferation and an increase in apoptosis, resulting in the reduction in size or the complete loss of an affected organ. However, Six1 may play additional roles during development, since *Six1 *knockout mice display significant defects in the differentiation of epibranchial progenitor cells into sensory ganglia [19], and since loss of *Six1 *in combination with loss of *Six4 *leads to defects in muscle cell differentiation and migration [23].

While expression of *Six1 *is low or absent in most adult tissues [11], it is re-expressed in a number of different human cancers including cervical [24], hepatocellular [25], ovarian [10], and breast cancer [11,26,27], as well as in alveolar rhabdomyosarcomas (RMS) [12,28], and Wilms' tumors [29]. *Six1 *has been extensively studied in breast cancer, where it is overexpressed in 50% of primary breast cancers, and 90% of metastatic lesions. These data suggest that Six1 may play an important role not only in the early stages of breast cancer, but also in the progression of breast cancer [11,27]. We recently demonstrated that Six1 overexpression in the mouse mammary gland is sufficient to induce tumorigenesis, leading to highly aggressive and invasive mammary tumors, many of which undergo an epithelial to mesenchymal transition (EMT) [30]. Interestingly, EMT has recently been associated with stem cell characteristics, and indeed we found that Six1 overexpression in mammary epithelial cells led not only to tumor formation, but also to an expansion of mammary stem/progenitor cells. In addition, we have recently shown that Six1 overexpression can induce metastasis in a xenograft model of breast cancer [31]. Since *Six1 *expression is often increased in cancers derived from tissues where it plays an important developmental role, including kidney and muscle [28,32], and since Six1 can increase the mammary stem/progenitor pool [30], we sought to determine if it is involved in the normal development of the mammary gland. In the present study, we have analyzed the expression pattern of *Six1 *during mammary gland development, and have explored the biological role of both inactivation and overexpression of *Six1 *in the development and function of the mouse mammary gland.

## Methods

### Experimental Animals

Mice were housed at the Center for Comparative Medicine at the University of Colorado Denver (UCD) Anschutz Medical Campus and treated in accordance with the NIH Guide to Humane Use of Animals in Research. All animal protocols were approved by the UCD-Institutional Animal Care and Use Committee (IACUC). *Six1*-deficient mice were previously described [16]. Two breeding pairs of heterozygous mice were used to initiate and maintain a breeding colony. Genotyping was performed using PCR with the following primer sets: the wild type allele was detected with WTm*Six1*F 5' GCG CCC GGG CCC GTG CGC CCC 3' (sense) and WTm*Six1*R 5' GCT TTC AGC CAC AGC TGC TGC 3' (antisense), and the mutated allele was detected with WTm*Six1*F and KO*Six1*R 5' TGC CCC AGG ATG TTG CCG TCC 3' (antisense). The embryos used in transplant experiments were first identified via phenotypic differences and EGFP expression of the whole embryo, after which PCR was used to confirm the genotype. C57Bl6/J mice were used for northern blot analysis and the developmental stages examined were E17.5, neonate (1 day post-partum), 5 and 10 week virgin, pregnancy (early: 5 days, middle: 12 days, late: 17 days), day 4 of lactation, and day 4 of involution. For embryonic and neonatal stages, each sample represents a pool of at least 15 animals. For all other stages, each sample represents a pool of 3 animals. For pregnancy, lactation, and involution, 10-week old mice were used and for involution studies, mice were allowed to lactate for 5 days prior to pup removal. qRT-PCR analysis was performed using *Six1 *knockout, heterozygote, and wild-type animals consisting of mammary glands harvested from 5 mice at embryonic day 18.5, representing a total of 15 animals for each genotype.

Hemizygous *MMTV-rtTA *(MTB) mice on an FVB background strain (obtained from L Chodosh) [33] were intercrossed with hemizygous TetSix animals to obtain the following genotypes: wild type, MTB, TetSix, and TOSix (bi-transgenic animals) as previously described [30]. Female littermates from the MTB and TOSix genotypes (6239 and 4922 lines) were mated and began treatment with water containing 5% sucrose and 2 mg/ml doxycyline (Sigma) at 12 weeks of age. A subset of TOSix animals were mated and treated with water containing 2% sucrose as a vehicle control as described [30]. Water was light-protected and changed weekly. Animals were sacrificed at pregnancy day 18 (P18) of first pregnancy for pregnancy timepoint and expression studies (qRT-PCR) and at lactation day 2 (L2) of first pregnancy for lactation timepoint. Animals were subjected to three rounds of pregnancy and were weaned of last litter at least four weeks before sacrificing to determine the state of regression of the mammary gland. Animals used for pup weight experiments were also subjected to three rounds of pregnancy. Litter sizes were normalized to eight pups, including any necessary fostering, and weights were taken each day from L1 to L21 during one of the three pregnancies, selected at random. Data were plotted based on average weight per pup and error bars reflect ± standard error of the mean (sem). Lactation and mammary regression experiments were only performed using dox-treated MTB and TOSix animals.

### RNA Isolation

For embryonic and neonatal stages, all mammary glands were taken and pooled as one sample. For later stages, the fourth mammary gland of female mice was used. Samples were flash-frozen in liquid nitrogen, and stored at -80°C. Total cellular RNA was isolated using Trizol™ reagent (Invitrogen) per manufacturer's protocol. The concentration and purity of RNA in each sample was determined by analyzing spectrophotometric absorption at 260/280 nm (Beckman).

### Northern Blot, qRT-PCR, and RT-PCR

Northern blotting was performed as described using probes made against full-length mouse *Six1 *and mouse *β-actin *[11]. For each timepoint, 20 μg of total RNA were electrophoresed in a formaldehyde-containing gel. The blot was stripped between hybridizations.

All RNA samples used for qRT-PCR analysis were isolated using the RNeasy Micro Kit (Qiagen), and were treated with DNase I in order to eliminate genomic DNA contamination. Two micrograms of total RNA per sample were used to generate cDNA using iScript per manufacturer's protocol (Bio-Rad). qRT-PCR for the HASix1 transgene was performed as previously described using TaqMan fluorescence probes [30]. qRT-PCR for mouse *Six *family members was performed using a model CFX96 instrument (Bio-Rad). Amplicons were detected using TaqMan fluorescence probes per manufacturer's protocol (IDT Integrated DNA Technologies). The primers and probes used for this study were as follows: *mSix1 *5' AAA GGG AGA ACA CCG AAA AC 3' (sense), 5' GGG GGT GAG AAC TCC TCT TC 3' (antisense), and 5' ACT CCT CCT CCA ACA AGC AGA ATC A 3' (probe), *mSix2 *GCC AAG GAA AGG GAG AAC AGC 3' (sense), 5' GCG TCT TCT CAT CCT CGG AAC 3' (antisense), and 5' ACC GAC TTG CCA CTG CCA TTG AGC G 3' (probe), *mSix4 *5'ACG TCC GAG ACC CAG TCC AAA AG 3' (sense), 5' TAT CTT AGC ATT TCC AAT TTG TTG C 3' (antisense), and 5' CCA GTA CCG AGG ATG AAT CCA GCA A 3' (probe), and *mSix5 *5' CCT AGT GAA TGG GAG CTT CCT G 3' (sense), 5' CCT TGG AGA CTG GGC TGT G 3' (antisense), and 5' CCA GCA GTG CTC CTC AAT GGT AGC C 3' (probe). Target genes were analyzed using standard curves to determine relative levels of gene expression, and individual cDNA samples were normalized according to the levels of *cytokeratin14 *(a marker of epithelial cells) using the following primers and probe: 5' GAG GAC TTG GTA GTG GAT TTG G 3' (sense), 5' AGC CCA TCA CCA ATA CCA C 3' (antisense), and 5' AAG ACC ACC ACC AAG ACC ACC ACC A 3' (probe). Analysis was performed using CFX manager version 1.0 (Bio-Rad). Statistics were generated using Prism 4 software (GraphPad) and p values determined using paired, 2-tailed student's *t*-Test. Error bars represent average ± SEM.

Before performing the RT-PCR reactions used to assess the expression of Six1 in knockout mice, all RNA samples were treated with DNase I (amplification grade, Invitrogen) for 10 min at room temperature in order to eliminate genomic DNA contamination. Two micrograms of total RNA per sample were used to generate cDNAs using Superscript II RNase H-reverse transcriptase (Invitrogen). The resulting cDNAs were subsequently amplified in a 50 μl reaction mixture containing 1 μM of each primer, 2 mM MgCl_2_, 0.8 mM dNTPs and 0.025 U/μl Taq DNA polymerase. Actin was used as a housekeeping control. Primer pairs used to amplify mouse *Six1 *(WT allele) were 5' GAA TCA ACT CTC TCC TCT GG 3' (sense) and 5' TTA GGA ACC CAA GTC CAC CA 3' (antisense), for EGFP (mutated allele) 5' CTG GTG ACC ACC CTG ACC TAC 3' (sense) and 5' TGA TCC CGG CGG CGG TCA CGA A 3' (antisense), and for actin 5' TAT CCT GAC CCT GAA GTA CC 3' (sense) and 5' GGT CAG GAT CTT CAT GAG GT3' (antisense). After denaturation for 2 min at 94°C, 30 cycles of amplification were performed using a thermocycler, followed by a final extension of 10 min at 72°C. The cycling parameters were: denaturation for 30 sec at 94°C, annealing for 1 min at 55°C, extension for 2 min at 72°C. After amplification, 20 μl of PCR product were electrophoresed on a 1% agarose gel containing 0.5 μg/ml of ethidium bromide.

### Histology and Immunofluorescence

Mammary glands were fixed in 4% paraformaldehyde overnight at 4°C, processed on a standard histology processor, embedded in paraffin, and cut into 3-5 micron sections. For histologic analysis, sections were stained with hematoxylin and eosin (H&E). For immunofluorescence, after dewaxing and hydration in graded alcohol solutions, the sections were treated with 10 mM citric acid, pH 6.0, in a microwave for 20 min, divided into 2 cycles. To prevent non-specific binding, the tissues were blocked with the M.O.M. mouse Ig blocking reagent (Vector Laboratories) in PBS for 16 h at 4°C. The tissues were then incubated with monoclonal mouse anti-GFP antibodies (Chemicon International), washed with PBS, and incubated with goat anti-mouse IgG conjugated with fluorescein (Calbiochem). Tissues untreated with primary antibodies were used as negative controls.

### Mammary Gland Transplantation

Whole mammary gland transplants were performed as described [34]. Briefly, pregnant female mice heterozygous for deletions in the *Six1 *gene were mated to heterozygous males and sacrificed at day 18 of pregnancy, at which time pups were removed. Animals were intraperitoneally injected with 100 mg/kg of BrdU two hours prior to euthanizing. Embryonic mammary glands from *Six1*^+/+ ^and *Six1*^-/- ^embryos were dissected with the aid of a stereoscopic microscope, and transplanted between the skin and abdominal wall (their normal position) of 5-week old C57Bl6/J-Rag1^-/- ^mice (Jackson Laboratories). The mammary glands were allowed to develop for 5 and 10 weeks. In some cases, mice were mated 10 weeks post-transplantation and the mammary tissues were harvested during pregnancy (5, 12 and 17 days). For each time-point, 3-5 transplant procedures per genotype were performed. The tissues were removed, fixed in 70% ethanol, embedded in paraffin, sectioned at 5-μm, and hematoxylin/eosin (H&E) stained.

### Cell Proliferation and Cell Death Assays

Cell proliferation was measured via BrdU incorporation using an immunohistochemical analysis kit (Amersham), whereas cell death assays were performed by terminal deoxynucleotidyl transferase-mediated dUTP nick end labeling (TUNEL) analysis using an apoptosis detection kit (Intergen).

## Results

### *Six1 *is dynamically expressed in the epithelium of the developing mammary gland

**Figure 1:**
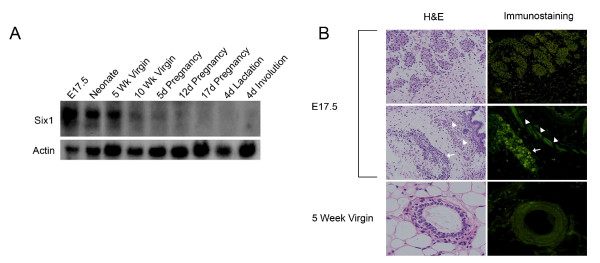
***Six1 *is dynamically expressed during mouse mammary gland development**. (A) Total RNA was isolated from different stages of mammary gland development and analyzed by northern blot. Note that *Six1 *is expressed at high levels until the fifth day of pregnancy when its level drops off significantly. (B) Mammary glands from *Six1 *heterozygote females were collected, fixed, and serial sections were stained with hematoxylin and eosin (H&E) or immunostained for EGFP, which replaced one allele of *Six1*. Arrows point to an island of mammary epithelial cells in the E17.5 embryo, which show intense positive staining for EGFP. Arrowheads point to developing muscles that are also EGFP-positive. Magnification, e17.5: ×40; 5 Weeks Virgin: ×100.

To examine whether *Six1 *is dynamically expressed in the developing mammary gland, therefore suggesting a functional role for the gene in this organ, we evaluated its expression by northern blot analysis using RNA isolated from mouse mammary glands encompassing different developmental stages. *Six1 *transcript levels were highest in embryonic day 17.5 (E17.5) embryos, and decreased progressively until day 5 of pregnancy, after which levels remained low during late pregnancy, lactation, and involution (Fig. 1A). This data confirms previously published qRT-PCR data [26].

While qRT-PCR and Northern blot data are suggestive of a dynamic pattern of *Six1 *expression in the mammary gland, they do not assess the cell type(s) in the mammary gland in which Six1 is expressed. Thus, to determine the spatial expression pattern of *Six1 *in the mammary gland, we utilized the previously described *Six1 *knockout mouse model [16]. Immunofluorescence analysis was performed on mammary glands isolated from *Six1 *heterozygote females, in which enhanced green fluorescent protein (EGFP) had been knocked into the *Six1 *locus [16]. Strong EGFP expression was observed in the invaginating epithelium in the mammary bud and in the developing muscle at day E17.5 (Fig. 1B). In addition, EGFP was detected in the ductal epithelial cells of the pubertal mammary gland (Fig. 1B). Thereafter, EGFP expression diminished in early pregnancy and reached a marginal level from mid-pregnancy through involution (data not shown). These data demonstrate that *Six1 *is expressed in the epithelial compartment of the mammary gland early during mammary development.

*Six1 *expression is increased in cancers derived from tissues where it plays an important developmental role, including Wilms' tumors (derived from the kidney) and rhabdomyosarcoma (derived from the muscle) [12,28,29]. *Six1 *is also overexpressed in breast cancers [11,27], and it has recently been shown to initiate invasive and aggressive mammary tumorigenesis and metastasis in both transgenic and xenograft mouse models [30,31]. Thus, we sought to determine whether Six1 plays a role in normal mammary gland development using the previously described *Six1 *knockout mouse model [16].

### Loss of *Six1 *expression does not affect mammary gland morphogenesis

Embryonic development of the mammary gland begins at day 12 with an invagination of the epidermis that results in a bulb-shaped assembly of epithelial cells, referred to as the mammary bud, surrounded by a layer of specialized mesenchymal cells. Around day 16, the mammary bud begins to elongate and to penetrate into the mesenchymal layer, initiating ductal branching morphogenesis [35]. Because *Six1 *is dynamically expressed throughout mammary gland development, and because of its recently described role in the expansion of mammary stem/progenitor cells [30], we reasoned that Six1 may be involved in the early stages of mammary gland development that result in the outgrowth of a ductal tree from the embryonic mammary bud.

To examine whether Six1 is indeed important for this early mammary gland expansion, we first analyzed glands from *Six1*^-/- ^embryos at days 15.5 and 18.5 of embryogenesis. To confirm the genotype of the mice used in this study, PCR analysis of tail DNA was performed using specific primer pairs to detect both the wild type and mutated alleles (Fig. S1A in Additional File 1). Expression of *Six1 *was examined in wild type, heterozygote and knockout mouse mammary glands by RT-PCR (Fig. S1B in Additional File 1), confirming the absence of *Six1 *in the mammary gland of the *Six1 *knockout mouse. E15.5 and E18.5 mammary glands from *Six1*^-/- ^mice showed completely normal morphogenesis compared with litter-matched wild type mice (data not shown). *Six1*^-/- ^embryonic mammary glands were smaller than *Six1*^+/+ ^mammary glands; however, *Six1*^-/- ^embryos were also smaller than litter-matched *Six1*^+/+ ^embryos. Thus it is not clear whether the reduction in size is intrinsic to the mammary bud itself.

**Figure 2:**
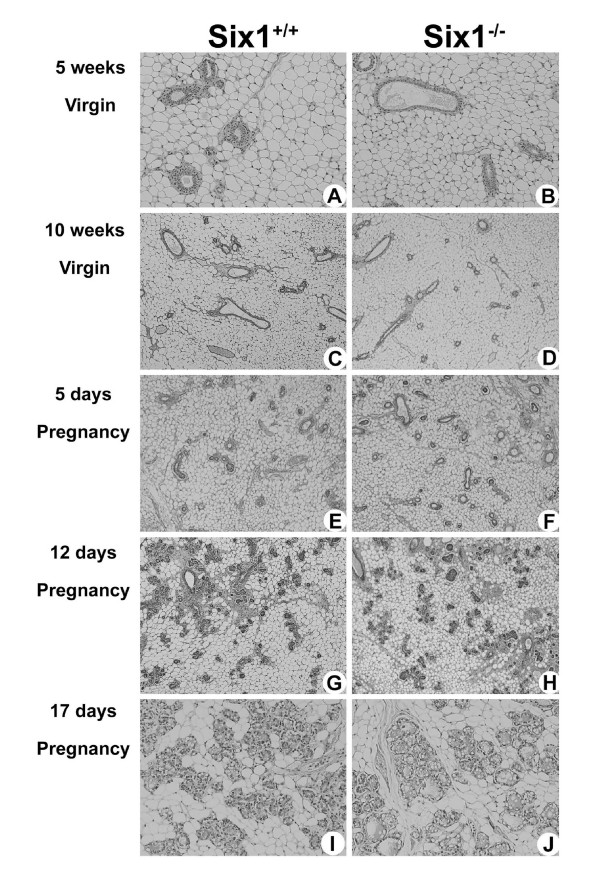
**Histological analyses of mammary glands transplanted into *Rag1*^-/- ^mice show that Six1 is not necessary for the normal development of the mammary gland**. Developmental stages are shown on the left, and the genotype of the animal from which the glands are derived is shown at the top. (A-B) At 5 weeks post-transplant, *Six1*^+/+ ^and *Six1*^-/- ^mammary glands were composed of epithelial ducts embedded in a soft connective tissue rich in fat. (C) A view of a *Six1*^+/+ ^mammary gland 10 weeks post-transplant shows a normal appearance. (D) The *Six1*^-/- ^mammary gland 10 weeks post-transplant demonstrates that the mammary ductal network is unaffected in the absence of *Six1*. Ten weeks after the transplant procedure, *Rag1*^-/- ^mice were mated and the transplanted mammary glands were analyzed at day 5 (E and F), day 12 (G and H) and day 17 (I and J) of pregnancy. Alveologenesis in pregnancy was not affected by *Six1 *deficiency.

Although no abnormalities were found in the mammary bud, we further sought to determine if disruption of the *Six1 *gene resulted in developmental defects in the mammary gland after embryogenesis; particularly since *Six1 *is still highly expressed in the neonatal and pubertal mammary gland, with more modest expression observed in the virgin adult and early pregnant mammary gland. Because *Six1*^-/- ^mice die shortly after birth, it was necessary to graft the E18.5 whole mammary gland into *Rag1^-/- ^*mice. The rate of successful mammary grafts was ~75%, independent of the genotype of the gland. Transplanted mammary glands from both wild type and knockout mice formed a normal branched ductal network at 5 and 10 weeks post-transplant (Fig. 2A, B, C, and 2D). Recipient mice were then mated to induce the differentiation of the mammary gland that occurs in pregnancy. *Six1*^+/+ ^glands are shown during early (Fig. 2E), middle (Fig. 2G), and late (Fig. 2I) pregnancy. Development of ducts and alveoli in *Six1*^-/- ^glands are normal (Fig. 2F, H, and 2J), as compared to *Six1*^+/+ ^glands. Alveoli in late pregnant glands from *Six1*^-/-^and *Six1*^+/+ ^animals were equivalent in size and a high proportion of their epithelial cells contained large cytoplasmic lipid droplets, indicating that secretory differentiation is also unaffected by *Six1 *deficiency [36]. Thus, surprisingly, no striking phenotype in mammary gland development was observed in the absence of *Six1*.

Since Six1 is known to control both cell proliferation and survival [7,8,15,16,26], we sought to determine if a deficiency in *Six1 *could subtly alter either mechanism by examining BrdU incorporation and TUNEL analysis, respectively, in wild type, and knockout mammary glands. BrdU- and TUNEL-positive cells were counted separately in the ductal and alveolar cells. There was no statistical difference between the number of BrdU-positive cells in *Six1*^-/- ^alveolar and ductal cells compared to *Six1*^+/+ ^cells (Fig. S2 in Additional File 2). Similarly, no difference in the number of TUNEL-positive cells was observed between *Six1*^+/+ ^and *Six1*^-/- ^transplants (data not shown). These results indicate that the absence of Six1 does not compromise mammary gland development on a gross level, and that proliferation and apoptosis are also not significantly affected by its absence.

### *Six2 *and *Six4 *transcript levels are elevated in *Six1*^-/- ^mammary glands

**Figure 3:**
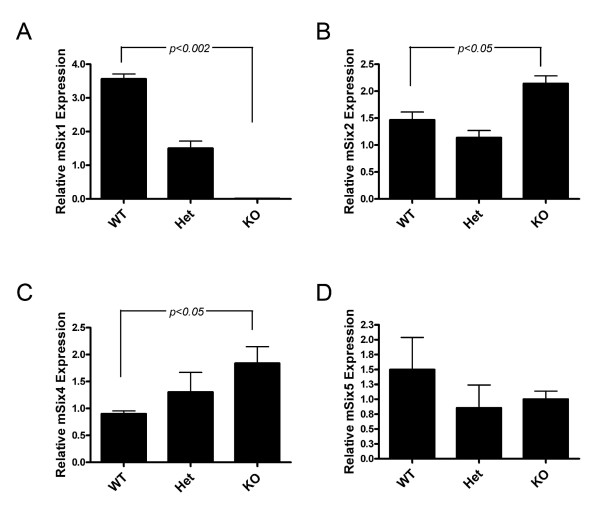
***Six1*-related family members *Six2 *and *Six4 *are upregulated in *Six1*^-/- ^mammary glands**. (A) qRT-PCR analysis confirms that *Six1 *transcript is not present in *Six1*^-/-^mammary glands. (B) *Six2 *and (C) *Six4 *RNA is elevated in *Six1*^-/- ^mammary glands. (D) *Six5 *transcript levels are not significantly different in *Six1*^-/- ^mammary glands as compared to wild-type controls. Data (A-D) was obtained from three pooled samples for each genotype, consisting of mammary glands harvested from 5 mice at embryonic day 18.5, representing a total of 15 animals for each genotype. Graphs are representative of two independent experiments. Error bars denote mean ± sem. P-values were obtained using a paired 2-tailed student's *t*-Test.

Several studies suggest that a compensatory mechanism exists amongst Six family members. Such studies include knockout mouse analyses that demonstrate that Six1 can compensate for Six4, since the *Six1/Six4 *double knockout animals have more profound phenotypes than do the *Six1 *or *Six4 *single knockout animals [23,37]. Additionally Six2, the family member most closely related to Six1, has been implicated in promoting a stem/progenitor phenotype in the kidney [38], suggesting that it may have similar functions to Six1, albeit in a different organ system. To determine if other Six family members may functionally compensate for the loss of Six1 in the mammary gland, we performed qRT-PCR analysis for all 6 *Six *family members in wild-type, heterozygous, and knockout genotypes, using mammary glands harvested at E18.5. Of the 6 *Six *family members, four were expressed in the E18.5 wild type mammary glands, including *Six1*, *Six2*, *Six4*, and *Six5 *(Fig. 3A, B, C, and 3D), whereas *Six3 *and *Six6 *did not amplify at detectable levels in any of the genotypes (data not shown). As expected, *Six1 *levels were reduced to about half of wild type levels in the *Six1 *heterozygote mammary glands, and were completely absent in the *Six1*^-/- ^mammary glands (Fig. 3A). In contrast, *Six5 *levels remained constant in all genotypes (Fig. 3D). Importantly, *Six2 *and *Six4 *transcript levels were both elevated in a statistically significant manner in *Six1*^-/- ^mammary glands (Fig. 3B and 3C). Together, these data suggest that other Six family members may compensate for the loss of Six1 function, particularly Six2 and Six4.

### Six1 overexpression in the mammary gland during pregnancy does not impair normal development

Although the results from our knockout studies suggested that Six1 is not *required *for mammary gland development, we hypothesized that *maintenance *of Six1 expression during pregnancy, lactation, and involution, when Six1 is normally downregulated, may inhibit differentiation of the gland. To test this hypothesis, we utilized a previously described inducible mouse model to overexpress Six1 specifically in the adult mammary gland [30]. Briefly, we crossed the MTB transgenic line expressing an MMTV-LTR driven reverse tetracycline transcriptional activator (*rtTA*) [39] with transgenic mice containing *Six1 *downstream of *tet *operator sequences (TetSix line), resulting in bi-transgenic offspring (TOSix). When the bi-transgenic animals are treated with water containing doxycycline (dox), rtTA is activated and able to bind to the *tet*-promoter, initiating transcription of *Six1*. Two transgenic lines, 4922 and 6239, were fully characterized [30] and chosen for the further experimentation.

**Figure 4:**
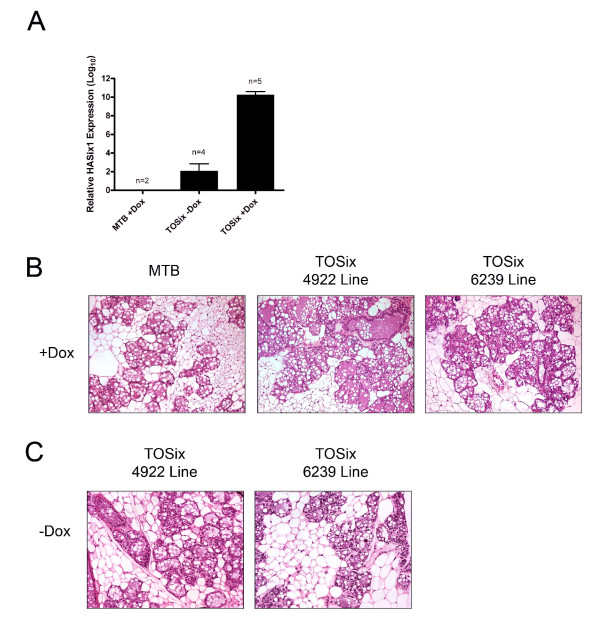
**Pregnancy occurs normally in TOSix animals**. (A) Real-time quantitative PCR (qRT-PCR), using transgene specific primers and probe, reveals that *HASix1 *is not expressed in the MTB control dox treated animals, but is expressed at low levels in the uninduced (-dox) mammary glands, and at high levels in the induced (+dox) mammary glands. Animals were sacrificed at pregnancy day 18 (P18) after beginning treatment with dox or sucrose vehicle-control at time of mating. Error bars denote mean ± sem. (B) H&E sections taken from MTB and TOSix mice. Animals were sacrificed at pregnancy day 18 (P18) after beginning treatment with dox at time of mating. (C) H&E sections taken from TOSix mice sacrificed at P18 after beginning treatment with sucrose-vehicle control at time of mating.

qRT-PCR data has shown that the *Six1 *transgene is expressed in aged, uninduced TOSix animals at low levels [30], demonstrating that the expression of *Six1 *is not completely dependent on dox induction in aged animals. Interestingly, tumor formation occurs at a higher frequency in the animals that express low levels of *Six1*, indicating that Six1 may act in a dose-dependent manner to induce mammary tumors [30]. Therefore, we sought to determine if *Six1 *is expressed in younger, uninduced TOSix animals, the focus of our mammary developmental studies, and whether *Six1 *was tightly controlled by dox in these animals. Twelve week old TOSix and MTB females were mated to wild-type males, and concurrently began continuous treatment with water containing 2 mg/ml dox or with sucrose as a vehicle control. At P18, females were sacrificed and their mammary glands excised for analysis. qRT-PCR data demonstrates that the *Six1 *transgene is expressed at low levels in the uninduced, sucrose-treated animals (-dox), but at much higher levels in the induced animals (+dox) (Fig. 4A). Thus, as was observed in the aged population, low levels of *Six1 *transgene expression can be observed in the mammary glands in the absence of dox induction. Therefore, we included animals from both cohorts in a number of the following studies to determine if the dose of Six1 has any affect on normal mammary development.

Hematoxylin and eosin (H&E) stained sections reveal that mammary glands taken from TOSix sucrose-treated or dox-treated animals display normal histology during pregnancy, and contain expanded alveoli that are producing lipid droplets, resembling the MTB control mammary glands (Fig. 4B and 4C). These data indicate that inappropriate Six1 expression, at either low or high levels, did not impact differentiation during pregnancy.

### Lactation and milk production occur normally in mammary glands overexpressing Six1

**Figure 5:**
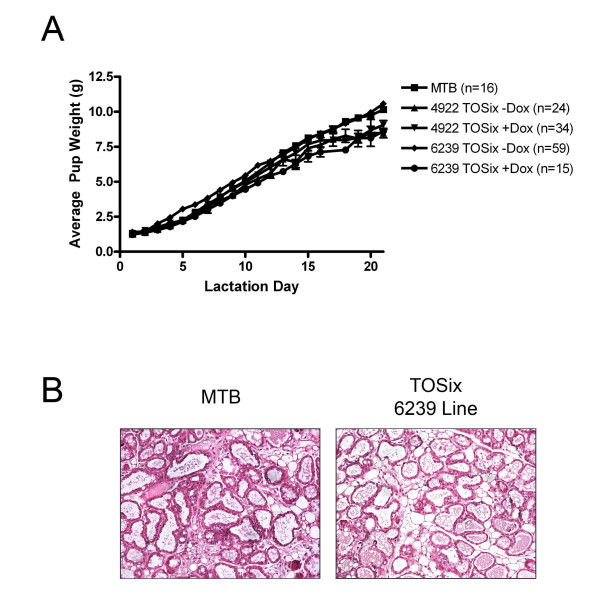
**Six1-overexpressing dams lactate normally**. (A) Average pup weight from TOSix and MTB litters, treated with dox or sucrose-vehicle as noted. Litters were normalized to 8 pups and weights were taken daily from lactation day 1 (L1) to L21. Error bars denote mean ± sem. n = average number of pups weighed (B) H&E stained sections taken from TOSix and MTB animals at L2. Animals began dox treatment at time of mating.

To determine if Six1 may affect normal lactation and milk production, pup weights were recorded for litters born to Six1-overexpressing dams. At 12 weeks of age, female animals from both the 4922 and 6239 lines began dox or vehicle-control treatment and MTB animals began dox treatment. All females were concurrently mated to wild-type males and were allowed to undergo three rounds of pregnancy. Litter sizes were normalized to eight pups and weights were taken each day from lactation day 1 (L1) to L21 during one of the three pregnancies, selected at random. No profound differences in pup weights were identified between litters born to TOSix +/- dox and MTB + dox dams, suggesting that TOSix dams lactate at a proficient level to meet the nutritional needs of their pups (Fig. 5A). Interestingly, we did note that a high percentage (approximately 80%) of 6239 TOSix +dox dams lost at least one of their litters during the three rounds of pregnancy as compared to 30-40% in MTB, 4922 line TOSix +/- dox, and 6239 line TOSix -dox animals (data not shown). However, this litter loss was inconsistent and did not appear to follow a clear trend. Some dams lost their litter only once during the three pregnancies, and in dams that lost litters, the loss didn't always occur in the same sequence (i.e. first, second, or third). We thus cannot attribute this finding to increased levels of Six1.

Although TOSix mothers capable of nursing did not exhibit any phenotype suggestive of lactation failure, we additionally analyzed mammary gland histology to determine any difference between the highest Six1-expressing and control mammary glands during lactation. For these experiments, 6239 line TOSix and MTB control virgin females were mated with wild-type males and concurrently began dox treatment. Resulting litters were normalized to eight pups, followed by sacrifice of the mother at L2. Histology of the mammary glands taken from TOSix (n = 3) and MTB (n = 3) dams reveal similar histology, including expanded ducts filled with milk (Fig. 5B). These results confirm the pup weight experiment results and suggest that mammary glands overexpressing Six1 are capable of fully differentiating and functionally lactating. We also performed this experiment using sucrose-treated animals and found that they exhibited normal mammary histology (data not shown).

### Six1-expressing mammary gland epithelium fully regresses following multiple pregnancies

**Figure 6:**
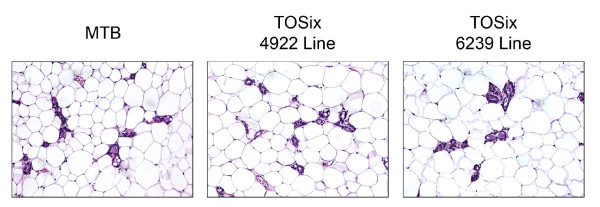
**Mammary glands overexpressing Six1 regress normally**. H&E sections taken from TOSix and MTB animals after 6 months of dox treatment (beginning at time of first mating) and after three rounds of pregnancy. Tissue was excised four weeks after weaning to ensure complete involution.

Since Six1 is known to play a pro-proliferative and anti-apoptotic role in numerous tissues [6-9], we hypothesized that persistent Six1 expression may prevent proper remodelling of the gland following pregnancy, thus contributing to the hyperplasia that eventually arises in these animals [30]. To test this hypothesis, TOSix females from both the 4922 and 6239 lines, as well as MTB controls began dox treatment at 12 weeks of age and were concurrently mated. This experiment was performed on the +dox TOSix group, and not the -dox TOSix group, as both groups were previously shown to equally exhibit hyperplasia after prolonged Six1 expression [30]. After the third pregnancy and 3 weeks of subsequent nursing, pups were weaned, and mothers were allowed to rest for four weeks to ensure complete involution before sacrifice. H&E stained sections reveal that the epithelium in mammary glands expressing Six1, taken from both the 4922 (n = 4) and 6239 (n = 2) transgenic lines, properly regresses, exhibiting similar histology to the glands taken from control MTB animals (n = 7) (Fig. 6). Whole mount analysis also revealed that TOSix mammary glands were fully regressed, similar to MTB mammary glands (data not shown).

## Discussion

Previous analysis demonstrated that *Six1 *expression is low to absent in the normal adult human mammary gland [11], and in agreement with this, its levels are very low in normal human breast epithelial cell lines [26]. In contrast, *Six1 *is overexpressed in both primary and metastatic breast tumors, as well as in several human breast cancer cell lines [11,26]. The molecular pathways involved in carcinogenesis often represent aberrations of normal processes that control embryogenesis [2]. *Six1 *is highly expressed in cancers derived from tissues in which it plays a fundamental role during embryogenesis [28,32], and recent work demonstrates that overexpression of Six1 leads to an expansion of mammary stem/progenitor cells [30]. These findings led us to reason that Six1 may play an important role in normal mammary gland development. Our results demonstrate that *Six1 *expression is indeed differentially regulated in the mammary epithelial compartment throughout mammary gland development, consistent with the hypothesis that Six1 plays a functional role in the development of the gland. Expression of *Six1 *correlates with several proliferative phases of mammary gland development, including those that occur throughout the course of ductal epithelial network development. *Six1 *transcript levels decline during pregnancy and lactation, when the mammary gland differentiates.

Studies in knockout mice demonstrate that Six1 is required for the proper development of the kidney, inner ear, thymus, nose, sensory neurons, muscle and lacrimal and salivary glands [7,8,14-16,19]. Multiple defects in *Six1*-deficient mice are associated with alterations in the proliferation, death, and fate of progenitor cells during embryogenesis [7,15,16,19]. In this study we demonstrate that, although *Six1 *is dynamically expressed in epithelial cells of the mammary gland during embryogenesis, no alterations in cellular proliferation or death are observed in *Six1*^-/- ^mammary glands, and these glands develop normally when transplanted into *Rag1*^-/- ^host mice.

It is well accepted that members of homeobox gene families exhibit functional redundancy [40,41], and such redundancy could explain the lack of a phenotype in *Six1*^-/- ^mammary glands. For example, redundancy has been observed in the mammary glands of mutant mice carrying deletions of paralogous homeobox genes *Hoxa-9*, *Hoxb-9*, and *Hoxd-9 *[41]. Single or double mutant lines disrupted for *Hoxa-9 *and *Hoxb-9 *showed no mammary gland phenotype, while animals simultaneously lacking *Hoxa-9*, *Hoxb-9*, and *Hoxd-9 *genes demonstrated marked hypoplasia of the mammary gland after parturition with gland morphology resembling that of a midpregnant animal. Studies suggest that a compensatory mechanism exists between *Six *family members. Interestingly, these experiments have revealed that Six1 can compensate for Six4 in muscle development, as the *Six1/Six4 *double knockout animals have more profound phenotypes than the *Six1 *single or *Six4 *single knockout, including failure of myogenic precursor cells to undergo delamination from the dermomyotome and to migrate to the limb bud, suggestive of a failure to induce an epithelial to mesenchymal transition [23]. These studies imply the existence of a unique redundant relationship between Six1 and Six4. Interestingly, our results reveal that both *Six2 *and *Six4 *are upregulated in the *Six1 *knockout mammary glands, suggesting that they may in fact both compensate for loss of Six1 function. Recent evidence demonstrates that Six2 regulates a stem cell population in the kidney [38], while data from our group has shown that Six1 may regulate the mammary epithelial stem cell population [30]. Given the expression pattern of *Six1 *throughout normal mammary development, Six1 may regulate expansion of stem/progenitor cells in early development, a function that could be compensated for by Six2. More studies need to be performed to better understand the redundant mechanisms within the *Six *family.

Although knockout studies revealed that *Six1 *is not required for mammary development, we postulated that its downregulation during pregnancy, lactation, and involution may be required for proper mammary gland differentiation. Six1 has pro-proliferative and pro-survival effects in a number of organ systems [6-9], and therefore we hypothesized that Six1 expression out of context during pregnancy, lactation, and involution, may in fact prevent proper differentiation and, ultimately, mammary function. Surprisingly, our results suggest that inappropriate expression of Six1 does not inhibit mammary gland differentiation during pregnancy, at least by gross histological analysis. Additionally, pups born to TOSix dams and control MTB dams thrive equally, as measured by pup weight, suggesting that lactation is also not influenced by the inappropriate expression of Six1. In confirmation of this finding, analysis of mammary gland histology during lactation further demonstrates that there is no significant defect in mammary gland function.

Finally, since Six1 plays a pro-survival role during development in other contexts [6-9], we hypothesized that Six1 overexpression would thwart proper mammary regression by preventing apoptosis and proper remodeling of the mammary gland during involution, particularly as we had previously shown that long term Six1 overexpression in multiparous mice leads to hyperplasia. Again, our studies suggest that regression ultimately occurs normally in young TOSix mice. It should be noted that we examined only whether involution had properly occurred 4 weeks after weaning. We did not test whether Six1 overexpression may delay involution by examining different time points throughout the early stages of involution. Nonetheless, our studies suggest that any effect of Six1 misexpression is overcome during the course of involution. As alluded to above, recent studies describing the role of Six1 in mammary tumor initiation describe a hyperplasia phenotype that arises in aged animals overexpressing Six1 [30]. Importantly, the hyperplasia phenotype displays features of precocious alveolar differentiation. While this suggests that Six1 may have some influence on pathways that control normal mammary differentiation, these phenotypes do not arise until animals are aged, and are not present in the younger animals used in this study. Six1-induced hyperplasia is thus most likely the result of an age-associated alteration in another pathway that cooperates with Six1 overexpression. As the data presented here show that regression of Six1-overexpressing mammary glands is complete following lactation, we do not consider the hyperplasia phenotype seen in aged animals as a "developmental" phenotype; rather, our data suggests that Six1 overexpression does not interfere with normal gland function or development.

It is possible that inappropriate expression of Six1 in later stages of development did not inhibit differentiation, at least in part, due to the lack of availability of its required cofactors Eya and Dach. Six1 is dependent on the Eya and Dach transcriptional cofactors to activate and/or repress transcription, as Six1 does not have an intrinsic activation or repression domain [15]. When Six1 and Dach are in a complex together, transcription of pro-survival and pro-proliferative target genes are repressed. However, if Eya is added to the complex, Six1 switches from a repressor to an activator [15]. Thus, it is possible that the availability of cofactors in the cell, as well as their stoichiometry with Six1, was not conducive to stimulating the pro-proliferative and anti-apoptotic functions of the protein, thereby removing the ability of aberrant Six1 expression to inhibit differentiation. Interestingly, overexpression of Six1 over long periods of time is sufficient to induce mammary tumors [30], suggesting that the appropriate levels of cofactors may be present for Six1 to properly activate transcription in a tumorigenic context. However, it remains to be determined whether this is indeed the case. Additionally, mammary development is an evolutionarily conserved process, and therefore multiple mechanisms most likely exist to override signals that would otherwise thwart proper development, such as Six1 overexpression. Together with results from the *Six1 *knockout mouse model, our data suggest that Six1 does not play a significant role in mammary gland development *in vivo*.

## Conclusions

In conclusion, we have shown that although *Six1 *is dynamically expressed in the mammary epithelium during mammary gland development, its expression is not required for proper embryonic or postnatal development, nor is its downregulation required for proper differentiation. While unexpected, our findings have significant implications for establishing whether Six1, which is overexpressed in a large percentage of breast cancers, is a reasonable chemotherapeutic target in a clinical setting, especially for those women diagnosed with breast cancer in their childbearing years. Of all the women diagnosed with breast cancer each year in the United States, 25% are diagnosed in their childbearing years. These women may desire future opportunities for pregnancy and lactation and therefore therapies that avoid the destruction of normal mammary tissue may prove advantageous in the clinic [42]. The fact that Six1 expression can induce mammary tumorigenesis and metastasis in mouse models, that its loss does not confer a mammary phenotype, and that it exhibits limited expression in the majority of normal adult tissues [11], makes it an exciting potential therapeutic target, as therapies directed against Six1 may inhibit breast tumorigenesis on multiple fronts while conferring limited side effects. Conditional knockout experiments in adult mice will enable us to determine whether Six1 is required in other adult tissues, and whether it truly could emerge as a viable therapeutic target.

## Competing interests

The authors declare that they have no competing interests.

## Authors' contributions

RDC carried out all transplant studies using the *Six1 *knockout mice, performed RT-PCR and northern blot analysis, as well as PCR for genotyping, completed immunostaining and cell proliferation and apoptosis analysis, and participated in drafting the manuscript. ELM performed all studies using the TOSix bi-transgenic mouse model, qRT-PCR for *Six *family members in *Six1 *knockout mammary glands, and participated in drafting the manuscript. VB assisted in transplant studies. KK provided the *Six1 *knockout mice. JLM assisted with the analysis of the mammary glands upon aberrant Six1 overexpression, and JJW assisted with analysis of embryonic mammary glands. HLF conceived the study, participated in its design and coordination, funded the study, and helped to draft the manuscript. All authors read and approved the final manuscript.

## Supplementary Material

Additional file 1 **Characterization of the *Six1*-deficient mice**. (A) PCR analyses of wild type (*Six1*^+/+^), heterozygote (*Six1*^+/-^), and knockout (*Six1*^-/-^) neonates. Tail DNA was isolated and PCR was performed using specific primer pairs as described in the Methods section. (B) RT-PCR analyses of mammary glands from *Six1*^+/+^, *Six1*^+/- ^and *Six1*^-/- ^at day E18.5. Absence of *Six1 *mRNA and expression of EGFP was confirmed in the *Six1*-deficient mammary gland. Click here for file

Additional file 2 **Loss of *Six1 *does not affect proliferation in the mammary gland**. Immunohistochemistry for BrdU was performed using mammary glands taken from *Six1*-/- and wildtype mice. Positive and negative cells were counted, both in ductal and alveolar structures, at d5 and d12 of pregnancy. Percent positive cells are represented. Click here for file
