# Supplementary figures and images for: Characterization of the Six1 homeobox gene in normal mammary gland morphogenesis
Source: BMC Dev Biol. 2010 Jan 14;10:4. doi: 10.1186/1471-213X-10-4 (PMC2823684; doi:10.1186/1471-213X-10-4)

A

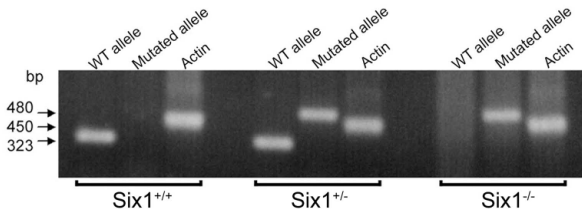

B

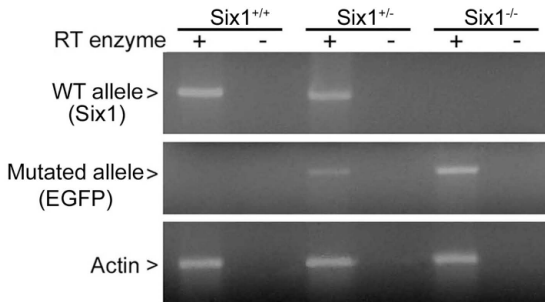

Supplement: Additional file 1 — Characterization of the Six1-deficient mice. (A) PCR analyses of wild type (Six1+/+), heterozygote (Six1+/-), and knockout (Six1-/-) neonates. Tail DNA was isolated and PCR was performed using specific primer pairs as described in the Methods section. (B) RT-PCR analyses of mammary glands from Six1+/+, Six1+/- and Six1-/- at day E18.5. Absence of Six1 mRNA and expression of EGFP was confirmed in the Six1-deficient mammary gland. [file 1471-213X-10-4-S1.PDF]

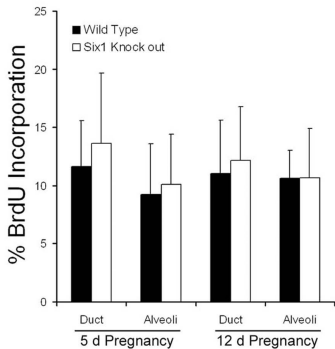

Supplement: Additional file 2 — Loss of Six1 does not affect proliferation in the mammary gland. Immunohistochemistry for BrdU was performed using mammary glands taken from Six1-/- and wildtype mice. Positive and negative cells were counted, both in ductal and alveolar structures, at d5 and d12 of pregnancy. Percent positive cells are represented. [file 1471-213X-10-4-S2.PDF]
